# Pramipexole Augmentation for Treatment-Resistant Unipolar and Bipolar Depression in the Real World: A Systematic Review and Meta-Analysis

**DOI:** 10.3390/life13041043

**Published:** 2023-04-19

**Authors:** Antonio Tundo, Sophia Betro’, Rocco de Filippis, Fulvia Marchetti, Daniele Nacca, Roberta Necci, Marica Iommi

**Affiliations:** 1 Istituto di Psicopatologia, Via Girolamo da Carpi, 1, 00196 Rome, Italy; 2 Dipartimento di Scienze Biomediche e Neuromotorie, Università di Bologna, 40126 Bologna, Italy

**Keywords:** pramipexole, treatment resistant depression, bipolar depression, unipolar depression, dopamine agonists, systematic review

## Abstract

Background: Pramipexole is a dopamine full agonist approved for the treatment of Parkinson’s disease and restless legs syndrome. Its high affinity for the D3 receptor and neuroprotective, antioxidant, and anti-inflammatory activity provides a rationale for the treatment of depression. In this paper, we review studies on the effectiveness and safety of antidepressant pramipexole augmentation in treatment-resistant depression. Methods: This comprehensive systematic review and meta-analysis of observational studies on pramipexole–antidepressant augmentation included patients with resistant unipolar and bipolar depression. The primary outcome measure was the treatment response, measured at the study endpoint. Results: We identified 8 studies including 281 patients overall, 57% women and 39.5% with bipolar disorder and 60.5% with major depressive disorder. The mean follow-up duration was 27.3 weeks (range 8–69). The pooled estimate of treatment response was 62.5%, without significant differences between unipolar and bipolar depression. Safety was good, with nausea and somnolence the most frequent side effects. Conclusions: The findings of this systematic review, needing further confirmation, show that off-label use of pramipexole as augmentation of antidepressant treatment could be a useful and safe strategy for unipolar and bipolar treatment-resistant depression.

## 1. Introduction

Major depressive disorder (MDD) and bipolar disorder (BD) are the most frequent psychiatric disorders, affecting about 20% of the general population in their lifetime [1]. They are associated with social withdrawal, functional and vocational impairment, medical morbidity, and premature death, including elevated suicide risk [2,3,4,5,6]. Major depressive episodes (MDEs) are the most common presentation of BD [7,8].

The antidepressant (AD) treatment of MDE comprises selective serotonin reuptake inhibitors (SSRIs), serotonin and noradrenaline reuptake inhibitors (SNRIs), monoamine oxidase inhibitors (MOAIs), tricyclic antidepressants (TCAs), and “other” antidepressants (mirtazapine, vortioxetine, amisulpride, trazodone, etc.). ADs are employed in monotherapy for unipolar depression [3,9,10] and combined with mood stabilizers or second-generation antipsychotics for bipolar depression without mixed features, rapid-cycling course, and treatment-emergent manic/hypomanic switch [3,6,11,12]. Still, a substantial subgroup of patients fails to respond to ADs. The results from the STAR*D study indicate that one in three patients with unipolar depression did not achieve symptomatic remission after several antidepressant trials [13,14], and in the STEP-BD study, patients with bipolar depression did not benefit from antidepressant treatment combined with mood stabilizers [15].

According to the European Medicines Agency (EMA), treatment-resistant depression (TRD) is defined as the failure to respond to treatment with two or more different ADs taken at adequate doses and duration [16]. Although the absence of consensus on TRD definition precludes a correct evaluation of the prevalence of this condition [17], there is a general agreement that it affects 20–30% of patients with unipolar [18] and bipolar [19] depression and that it is associated with increased suicide risk, poor prognosis, physical health decline, and increased health care utilization [20,21,22]. The concept of treatment resistance originates from the context of MDD and the definition of bipolar TRD has been extrapolated from the definition of unipolar TRD [23]. This definition has been supported by data in an unambiguous way [24] since the efficacy and safety of ADs for bipolar depression are highly controversial [25]. However, ADs are commonly used in clinical practice [26,27] and their use, as mood stabilizers and/or SGA augmentation, is recommended in specific conditions by expert consensus [11] and guidelines [3,6,24,28]. Moreover, in studies on bipolar depression, resistance is defined as the lack of remission after two adequate trials with standard ADs, so the limitation of this definition should be taken into account when the results of these studies are evaluated.

Different pharmacological strategies have been proposed for TRD [29,30]; one of the best established [17,31,32] and most employed in clinical practice [33] is the addition to the current AD of a non-antidepressant medication, mostly lithium salts, thyroid hormone, or a low dose of second-generation antipsychotics, mostly aripiprazole and quetiapine. Regulatory agencies, in consideration of the available evidence [34,35], recently approved Esketamine nasal spray, an N-methyl-D-aspartate receptor antagonist, in addition to SSRIs or SNRIs for TRD.

Given the severity of TRD and the uncertain efficacy of available augmentation strategies [33], new and more effective drugs for AD augmentation are an unmet need in the field.

More data are needed specifically for bipolar TRD because most of the studies on the treatment of TRD include only patients with unipolar TRD [36].

Old and new research in human and animal models, using neuroimaging, pharmacological, and electrophysiological methods, suggests the possible role of dopamine (DA) dysfunctions in mood disorders [19]. More in detail, the available evidence indicates that: (a) levels of DA are decreased in depression and increased in mania [37,38]; (b) D2 receptor binding is increased in the striatum in depression [39]; (c) striatal DA transporter is increased (leading to reduced DA functioning) in depression and striatal D2/3 receptor availability is increased (leading to increased DA neurotransmission) in mania [40]; (d) different DA receptors, mostly D1-D2 heterodimers, and their distribution in different brain regions could be involved in the etiology of depression [41]; (e) the highly effective antidepressant treatments (tricyclic and monoamine oxidase inhibitor antidepressants and electroconvulsive therapy) have as a common effect the increased expression of the D3 receptor in the nucleus accumbens [42]; (f) mesolimbic DA neurons seem to be linked to some nuclear symptoms of depression such as loss of motivation and motor retardation [43]; (g) dopamine agonists are effective in animal models of depression [44,45,46] as well as an antidepressant-like and anxiolytic-like effect in rats [47]; and (h) compared with healthy individuals, depressed patients with anhedonia have significantly lower DA transporter binding in PET imaging studies [48].

Currently used dopamine agonists are classified into ergot alkaloids, which can cause rare but serious adverse events such as valvular heart disease, and non-ergot alkaloids, which do not have such effects on cardiac valves [49]. One of the latter is pramipexole, a full dopamine agonist with a higher affinity for the D3 receptor than for the D1, D2, and D4 receptors, which is FDA-approved for the treatment of Parkinson’s disease and restless legs syndrome [50,51]. Its marked selectivity for D3 receptors, which are in high concentrations in mesolimbic areas and are implicated in mental processes related to emotion and mood [43,52], and its neuroprotective, antioxidant, and anti-inflammatory activity [53,54,55] provide a rationale for the treatment of depression. The results of a recent experimental investigation further support the hypothesis of potential antidepressant activity of pramipexole, showing that its subacute administration (12–15 days at a peak daily dose of 1.0 mg) in healthy volunteers modifies neural responses to emotional information similarly to that of traditional antidepressants [56].

This drug is bound to plasma protein to a very low (<20%) extent, does not inhibit CYP isoenzymes 1A2, 2C9, 2C19, 2E1, 3A4, or 2D6, is not appreciably metabolized by CYP isoenzymes, and is secreted by the renal tubules (90%). The information leaflet (https://www.accessdata.fda.gov/drugsatfda_docs/label/2018/020667s036lbl.pdf (accessed on 31 December 2022) does not report any potential interaction of pramipexole with lithium, valproate, and carbamazepine and points out a possible additive sedative effect with antidepressants, antipsychotics, and benzodiazepines. Furthermore, it does not report a risk of metabolic changes or QTc interval prolongation.

In clinical practice, some studies have evaluated the efficacy and safety of pramipexole as an antidepressant in patients with Parkinson’s disease and patients with mood disorders.

Regarding the patients with Parkinson’s disease, a meta-analysis of 18 randomized controlled trials showed that the improvement of depressive symptoms in the pramipexole treatment group was significantly higher than in the control group without differences in the side effects rate [57].

Regarding the patients with mood disorders, a meta-analysis including 13 studies published up to December 2018 (5 randomized clinical trials and 8 observational studies) and 504 patients (362 with unipolar depression and 142 with bipolar depression) [58] estimated that pramipexole treatment had a 52.2% response rate in the short term and a 62.1% response rate in the long term and a 36.1% remission rate in the short term and a 39.6% remission rate in the long term. In randomized clinical trials (RCTs), the response rate to pramipexole was superior to that of a placebo (mostly in the bipolar depression subgroup) and similar to that of SSRIs. Acceptability and tolerability were good, with nausea being the most frequent side effect.

Although encouraging about the antidepressant properties of pramipexole, this evidence does not allow us to evaluate the effectiveness of pramipexole as AD augmentation in patients with TRD in the real world because of the heterogeneity of the studies included in the review. In fact, 2 of the 13 studies did not include patients with TRD and 5 of the 13 studies evaluated the antidepressant property of pramipexole as monotherapy and not as augmentation of traditional AD.

### Aims of the Study

This study’s aim is to conduct a systematic review and meta-analysis of observational studies on the effectiveness and safety of pramipexole AD augmentation in patients with unipolar and bipolar TRD.

## 2. Materials and Methods

### 2.1. Literature Search

The PRISMA method [59] was followed in the literature search.

Specifically, PubMed/MEDLINE, Cochrane Central Register of Controlled Trials (CENTRAL), and Embase databases were searched to identify peer-reviewed articles on the effectiveness and safety of pramipexole for major depressive episodes in unipolar and bipolar depression. The search string used the following terms: mood disorders, depression, affective symptoms, affective disorder, mood disorder, bipolar, mania, manic, hypomania, pramipexole, and dopamine agonists. We searched for ongoing and unpublished studies via Internet searches on ClinicalTrials.gov (www.clinicaltrials.gov (accessed on 31 December 2022)) and on the World Health Organization (WHO) International Clinical Trials Registry Platform (ICTRP) (apps.who.int/trialsearch/ (accessed on 31 December 2022).

No beginning date and no language restrictions were applied, and the last publication date was 31 December 2022.

### 2.2. Inclusion and Exclusion Criteria

Retrospective or prospective observational studies and case series using pramipexole as an AD augmentation strategy for treatment-resistant unipolar and bipolar MDE were included.

Randomized clinical trials, observational studies using pramipexole as monotherapy, and observational studies including patients without TRD were excluded.

### 2.3. Population

Male and female patients aged ≥18 years with a primary diagnosis of MDD or BD were included. Studies including patients with other comorbid psychiatric disorders, suicidal thoughts, or serious medical illnesses were not excluded.

### 2.4. Outcome Measures 

The primary effectiveness outcome measure was the treatment response, defined as a ≥50% reduction from the baseline of the Montgomery–Asberg Depression Rating Scale (MADRS) [60], Hamilton Depression Rating Scale (HDRS) [61], or Clinical Global Impression (severity) [62].

The secondary effectiveness outcome measure was remission, defined as reaching a sub-threshold score on the depression scale used in the specific study (for instance, a score of HDRS-17 ≤ 7) at the endpoint.

If neither of these three scales were used, we considered other clinician-rated or self-report scales.

### 2.5. Safety Measures

The safety measures were: (1) tolerability, i.e., the number of patients who discontinued the study due to side effects and the number and types of side effects (2) acceptability, i.e., the number of patients who discontinued treatment for any reason; (3) (hypo)mania onset; and (4) suicide attempt.

### 2.6. Data Extraction

Two researchers (S.B. and R.d.F.) read each article and evaluated the completeness of the data extraction independently. A structured data retrieval form was designed to ensure consistency of appraisal for each study. The data included study characteristics (such as lead author, publication year, and journal), participant characteristics (age range, setting, and diagnosis), intervention details (dose range and mean dose of study drugs), and outcomes of interest. The data were extracted from the manuscript and the tables, while PlotDigitizer 2.6. [63] was used to extract the data from the figures. Information on primary and secondary outcomes was extracted by the same two researchers, and disagreements were resolved in a consensus meeting with a third researcher (AT).

### 2.7. Statistical Analysis 

A random-effects meta-analysis was performed to obtain the overall pool estimates with a 95% confidence interval (95% CI) of primary, secondary, and safety outcomes; the pool estimates were also estimated and compared between bipolar and unipolar depression.

Between-study heterogeneity was tested by Cochran’s Q test and measured with the I2 statistic, which represents the percentage of variance in the estimated effects due to heterogeneity rather than chance and ranges from 0 to 100. An I^2^ statistic > 50% was considered indicative of significant heterogeneity.

Forest plots were used to graphically depict the estimates with 95% CI for individual studies and pooled results. All statistical analyses were performed with R version 4.2.0 using the meta and metafor packages.

## 3. Results

### 3.1. Selected Studies

**Figure 1:**
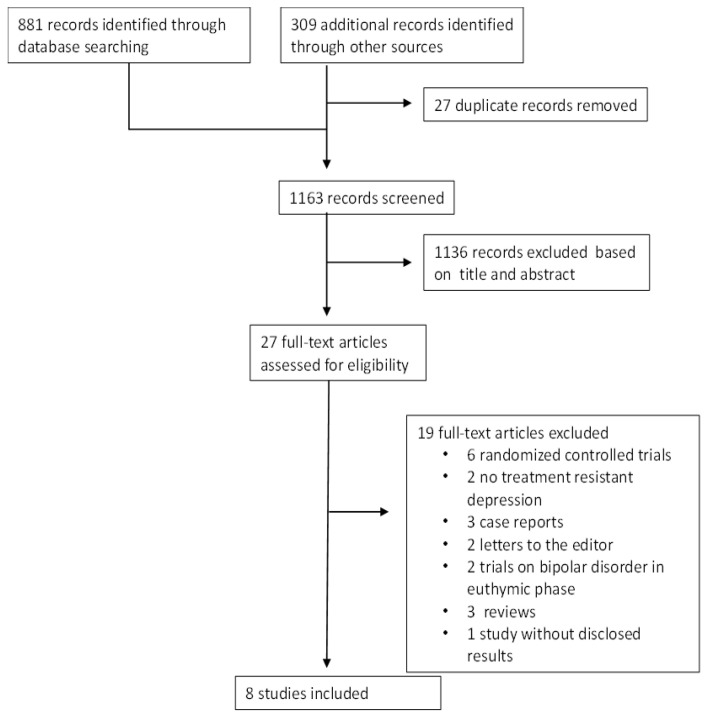
Flowchart of the selected studies.

The literature search generated 881 records and another 309 records were identified from trial registers. After duplicate removal through EndNote, 1163 titles and abstracts were initially assessed; 1136 articles were excluded from the title and abstract, and 27 articles were retrieved in full text. Nineteen studies were excluded for the following reasons, three were RCTs including patients without TRD [64,65,66], three were RCTs [67,68,69], two included patients without TRD [70,71], three were case reports [72,73,74], two were letters to the editor [54,75], two were trials conducted on individuals with bipolar disorder in the euthymic phase [76,77], three were reviews [31,58,78], and one was a clinical trial on individuals with bipolar disorder treated with quetiapine extended-release and pramipexole, but the results were not disclosed [79] (see flow chart, Figure 1).

Finally, 8 studies (281 participants) were included in the present systematic review [49,80,81,82,83,84,85,86]. One study considered only unipolar patients [81], one study considered only bipolar patients [83], while one study did not differentiate the results with respect to diagnosis [80].

### 3.2. Study Characteristics

The main study characteristics are listed in Table 1.

Three studies were prospective observational studies, two were open-label trials, two were chart reviews, and one was a case series. Six studies used the DSM-IV or the subsequent editions for diagnosis [49,80,81,82,84,86], one used DSM-III-R [83], and one did not report which diagnostic criteria were used [85]. One hundred and seventy patients (60.5%) had a diagnosis of MDD and one hundred and eleven (39.5%) had a diagnosis of BD.

The mean study sample size was 35 (range 10–116), 57% were women, the mean age was 49.6 years, and the mean age at onset was 33.6 years (76 did not report this information).

All of the studies measured the outcomes using standardized scales, such as HDRS, MADRS, or CGI, except for one [85]. Pramipexole was used at flexible doses and the mean maximum dose was 1.24 mg (range 0.69–2.18). The median duration of treatment was 27.3 weeks (range 8–69).

### 3.3. Outcome Measures

- Treatment response

Overall, significant heterogeneity was found across the eight studies (I2 = 62.7%, 95% CI 19.8–82.7%; *p* = 0.009) and the pooled treatment response estimate was 62.5% (95% CI 52.3–72.7%).

**Figure 2:**
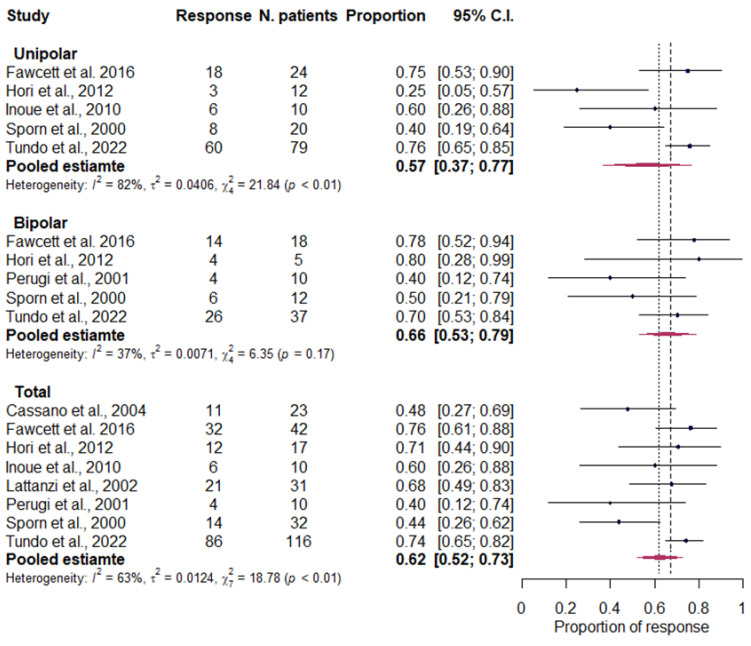
Forest plot with the total pooled estimates of treatment response and by diagnosis. Cassano et al., 2004 [80]; Fawcett et al., 2016 [85]; Hori et al., 2012 [49]; Inoue et al., 2010 [81]; Lattanzi et al., 2002 [82]; Perugi et al., 2001 [83]; Sporn et al., 2000 [84]; Tundo et al., 2022 [86].

In the analysis stratified by diagnosis, significant heterogeneity was found among the studies related to unipolar depression (I2 = 81.7%, 95% CI 57.6–92.1%). The meta-estimate of treatment response was 56.7% (95% CI 36.7–76.6%) for unipolar depression and 66.0% (95% CI 53.1–78.8%) for bipolar depression (Figure 2). The response to treatment did not differ between the two disorders (*p* = 0.443).

b. Treatment remission

**Figure 3:**
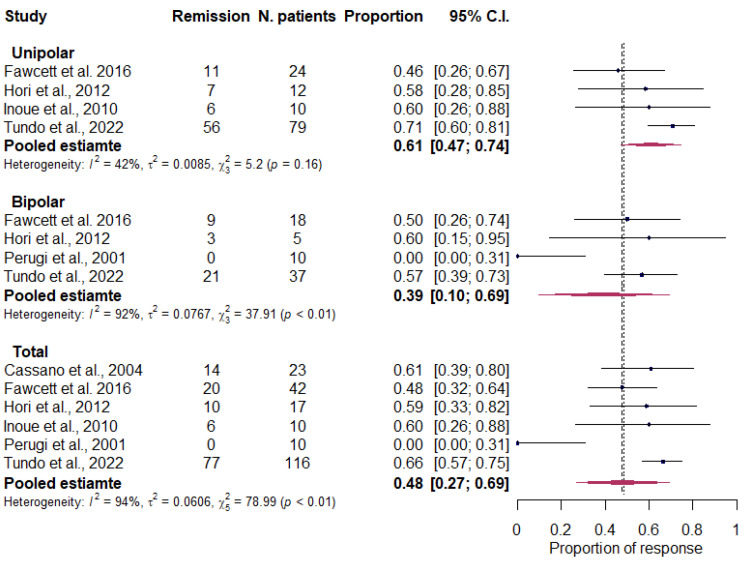
Forest plot showing the total pooled estimates of remission and by diagnosis. Cassano et al., 2004 [80]; Fawcett et al., 2016 [85]; Hori et al., 2012 [49]; Inoue et al., 2010 [81]; Perugi et al., 2001 [83]; Tundo et al., 2022 [86].

The six studies reporting remission data (218 participants) were significantly heterogeneous (I2 = 93.7%, 95% CI 88.9–96.4%; *p* < 0.001), with a total pooled estimate of remission of 48.1% (95% CI 27.0–69.3%) (Figure 3).

Although the pooled proportion of remission in unipolar depression was higher than that in bipolar depression (60.8% vs. 39.4%), no significant difference was found between the two groups (*p* = 0.200).

### 3.4. Safety Measure

- Drop-outs due to any adverse event

The estimated pooled proportion of dropouts due to adverse events was 11.4% (95% CI 5.0–17.8%), based on seven studies reporting this information (271 participants) (Supplementary Table S1).

The proportion of dropouts due to adverse events did not differ between bipolar (10.1%, 95% CI 3.1–117.2%) and unipolar depression (6.4%, 95% CI 2.3–10.5%) (*p* = 0.372).

Common side effects (incidence > 1/100, <1/10) were nausea (*n* = 21, 7.4%), somnolence (*n* = 13, 4.6%), agitation (*n* = 10, 3.5%), tremors (*n* = 6, 2.1%), dry mouth (*n* = 5, 1.7%), dizziness upon standing (*n* = 5, 1.7%), irritability (*n* = 5, 1.7%), increased sex drive (*n* = 5, 1.7%), and insomnia (*n* = 4, 1.4%). Uncommon side effects (incidence > 1/1000, <1/100) were headache, visual hallucination (*n* = 2, 0.7% each), delirium, ataxia, anxiety, itching, difficulty urinating, vivid dreams, tics, increased appetite, and word findings difficulty (*n* = 1, 0.3% each).

b. Drop-outs due to any reason

Overall, when pooling the seven studies with data on drop-outs due to any reason (271 participants), the proportion of dropouts was 29.7% (95% CI 16.2–43.3%). For the bipolar group, the pooled proportion of dropouts was 12.8%, while in the unipolar group, it was 20.4%; however, the difference was not significant (*p* = 0.424) (Supplementary Table S2).

c. (Hypo)mania onset

(Hypo)mania onset was uncommon in both groups (no difference between the groups, *p* = 0.098); indeed, the global pooled proportion of (hypo)mania was 1.1% (95% CI 0–2.0%) (Supplementary Table S3).

d. Suicide attempt

No patients attempted suicide.

## 4. Discussion

To the best of our knowledge, this is the first systematic review of observational studies to evaluate the effectiveness and safety of pramipexole as AD augmentation on treatment-resistant unipolar and bipolar depression. We focused on these studies for multiple reasons. Observational studies include patients with psychiatric and physical comorbid disorders and with suicidal thoughts, often excluded from RCT, providing evidence on response/remission rates and adverse events associated with treatment in the real world [87]. Furthermore, effective dosage and titration of pramipexole as AD augmentation are not yet clearly defined and the flexible prescription in observational studies, based on the clinical judgement of the prescriber according to the tolerability and therapeutic efficacy, provide valuable information on the use of the drug that maximizes tolerability and effectiveness [85,86]. Notably, the present study includes 7 of the 13 studies included in a previous review [58] and a study published later [86]. Five studies of previous revision were excluded because of RCTs and one because it was conducted on patients with non-treatment-resistant depression.

This systematic review and meta-analysis suggest that the augmentation of traditional antidepressants with pramipexole could be an effective strategy for TRD.

The pooled estimates of overall response (62.5%) and remission (48.1%) with pramipexole augmentation are close to the higher boundary of the range of response and remission rates reported in the literature for aripiprazole augmentation (18.5% to 60% and 7.4% to 54%, respectively) [30,88], the strategy for TRD with the strongest evidence of efficacy [31,89,90] and extensively used in clinical practice [33].

Furthermore, response and remission rates for pramipexole are quite higher than those reported for the treatment of unipolar non-resistant depression with ADs (52.9% and 32.6%, respectively) [91].

Regarding the pre-planned subgroup analyses, the response rate was quite similar for individuals with unipolar and bipolar depression (66.0% and 56.7%, respectively), while the remission rate was higher for unipolar than for bipolar individuals (60.8% and 39.4%, respectively), although the difference failed to reach the statistical significance because of the small sample size. This result did not confirm the finding of a previous review [90] showing a significantly higher response rate to pramipexole than to a placebo in bipolar but not in unipolar treatment-resistant and non-resistant depression (RCT studies). Considering the limited information on this topic, evidence supporting the use of pramipexole augmentation for bipolar TRD as well as for unipolar TRD could be useful for clinicians engaged in the routine clinical management of bipolar patients with TRD. This result should be interpreted with caution because ADs use in patients with bipolar depression is controversial and the definition of bipolar TRD is not clearly supported by the data.

The topic needs further investigation in larger samples.

Notably, one study included in the present review [86] compared response and remission rates in patients with (*n* = 32) and without (*n* = 84) chronic cerebrovascular comorbid disease showing no statistical differences between the two groups. This evidence, needing further confirmation, could be clinically relevant because patients with depression and chronic cerebrovascular diseases are often poor respondents to ADs [92,93], and data on the effectiveness of lithium or aripiprazole AD augmentation for patients with TRD comorbid with chronic cerebrovascular diseases are very limited [94,95].

Our findings indicate that pramipexole augmentation is a safe strategy for TRD with a drop-out rate due to side effects of 11.4% and due to any reason of 29.7%. Furthermore, during the trials, no patients attempted suicide and only a very low number of patients developed (hypo)mania.

Although the profile of side effects was similar to that usually reported for patients with Parkinson’s disease (https://www.drugs.com/sfx/pramipexole-side-effects.html (accessed on 31 December 2022), the frequency of these effects was lower. In patients who did not drop out, side effects often resolved spontaneously or after reducing the pramipexole dose [85,86]. Although the information leaflet reports a potential interaction of pramipexole with antidepressants, antipsychotics, and benzodiazepines (sedative effect), no study included in the present review reports drop-out or side effects due to this interaction.

Transient visual hallucination and confusion occurred respectively in 2 (6.2%) and 1 (3.1%) of the 32 old patients with chronic cerebrovascular comorbid disease included in Tundo et al.’s study [86]. Although the rates of these side effects were lower than that reported in patients with Parkinson’s disease (17% and 10%, respectively) (https://www.drugs.com/sfx/pramipexole-side-effects.html (accessed on 31 December 2022), the authors suggest prescribing pramipexole with caution in elderly patients with comorbid chronic cerebrovascular conditions and careful monitoring of delirium or psychotic symptoms.

Notably, the studies included in this systematic review report a very low rate of hypersexuality and no cases of others impulse dyscontrol (gambling and compulsive shopping), which are common in patients with Parkinson’s disease receiving pramipexole [96]. This result is in line with those of a previous meta-analysis on pramipexole including observational and RCT studies in which pramipexole is used as monotherapy or as AD augmentation [58]. The different susceptibility to impulse dyscontrol between patients with mood disorders and with Parkinson’s disease might be due to different neurobiological dysfunctions underlying these two diseases [97,98], although we cannot exclude that the relatively small sample size of patients with TRD treated with pramipexole precludes the possibility to detect these adverse events.

Overall, the safety of pramipexole augmentation is comparable to that reported in the literature for traditional antidepressants employed for the treatment of unipolar non-resistant depression and for aripiprazole augmentation, the strategy for TRD with the strongest evidence of efficacy.

In fact, the tolerability and acceptability of pramipexole augmentation were quite similar to that of traditional antidepressants for the treatment of unipolar non-resistant depression (drop-out rate due to any type of side effects 11.4% and 10.4%, respectively; drop-out rate for any reason 29.7% and 26.4%, respectively) [91] and tolerability was slightly better than that reported for aripiprazole augmentation (drop-out rate due to any side effect 11.4% and 23.7%, respectively) [99]. We found only six studies reporting the acceptability of aripiprazole augmentation [89] showing that the drop-out rate due to any reasons for this augmentation is lower than that of pramipexole augmentation (14% and 29.7%, respectively), but the difference in follow-up duration, shorter for aripiprazole than for pramipexole (6 and 27.3 weeks median duration, respectively) could explain the difference in acceptability.

The main limitation of the present review is the restricted number of studies and participants. This limitation may have contributed to the lack of statistical significance in subgroup comparisons. Other limitations of the study were the high variability of dosing and the time points at which outcomes were collected, and the lack of a critical appraisal of the articles.

Furthermore, significant heterogeneity between the studies was observed that could not be controlled using covariates in a meta-regression because of the limited information available in the studies.

## 5. Conclusions

In conclusion, evidence from real-world studies suggests that adding pramipexole to traditional ADs could be an effective treatment option for patients with TRD. Pramipexole augmentation appeared to be safe, with nausea and somnolence being the most frequent side effects.

Should the findings of the current review be confirmed by high-quality and well-designed RCTs, clinicians would have a further augmentation strategy for TRD with a different side effects profile from the current strategy with the best evidence of effectiveness (SGA augmentation), without QTc prolongation, metabolic, and akathisia risk. Thus, clinicians may choose the most suitable treatment for each patient according to his/her medical condition and preference.

We reiterate that the use of pramipexole as AD augmentation is off-label and its prescription is currently allowed only to centers specialized in TRD [9] and carefully selected patients without current or previous episodes with psychotic symptoms because pro-dopaminergic agents could induce psychosis in vulnerable individuals [100].

Pramipexole use requires special precautions. The dosage must be gradually increased to reduce the risk of side effects, mostly nausea, and gradually escalated to avoid the risk of dopamine withdrawal syndrome. Patients and their family members must be instructed to report the occurrence of any potentially dangerous side effects such as lethargy, hypersexuality, gambling, compulsive shopping, and, mostly in old patients with chronic cerebrovascular comorbid diseases, psychotic symptoms or delirium. Although the preliminary data suggest the safety of pramipexole in the treatment of bipolar depression, evidence from further studies is needed to confirm the low risk of mixed features, (hypo)manic switch, or rapid cycling course development in patients with bipolar depression.

## Figures and Tables

**Table 1:** Characteristics of the observational studies included in the meta-analysis.

Study	Design	Participants (*n*)	Females	Age Mean, Years (SD)	Age at Onset Mean, Years (SD)	Diagnoses	Pramipexole Mean Maximum Dose	Trial Duration (Weeks)	Outcome Measure	Response Criteria	Remission Criteria
Cassano et al., 2004 [80]	Prospective	23	69.5%	52.8 (12.5)	35.1 (16)	11 MDD; 12 BD	0.99	48	LIFE	Not reported	Depression score ≤ 2
Hori et al., 2012 [49]	Open-label trial	17	58.8%	36.2 (9.2)	28.1 (7.6)	12 MDD; 5 BD	1.6	12	HDRS	>50% total score reduction	Total score ≤ 7
Inoue et al., 2010 [81]	Open-label trial	10	40%	43.7 (11.4)	39.6 (11.5)	10 MDD	1.3	8	MADRS	>50% total score reduction	Total score < 10
Lattanzi et al., 2002 [82]	Prospective	31	67.7%	53.7 (13.5)	32 (15.3)	14 MDD; 17 BD	0.95	16	MADRS	>50% total score reduction	Not reported
Perugi et al., 2001 [83]	Retrospective	10	60%	55 (15.9)	34.2 (14.8)	10 BD	1.23	17.6	CGI	Improvement score = 2	Improvement score = 1
Sporn et al., 2000 [84]	Retrospective	32	53.1%	41.5 (14)	22.3 (10.8)	20 MDD; 12 BD	0.69	24.4	CGI	Improvement score ≤ 2	Not reported
Fawcett et al., 2016 [85]	Case series	42	50%	53.97 (13)	NR	24 MDD; 18 BD	2.18	69	Clinical	Clinical assessment	Clinical assessment
Tundo et al., 2022 [86]	Prospective	116	56%	62.2 (13.3)	44.4 (17.9)	79 MDD; 37 BD	1.03	24	HDRS	>50% total score reduction	Total score < 7

Abbreviations: BD = bipolar disorder; MDD = major depressive disorder.

## Data Availability

Not applicable.

## Supplementary Materials

The following supporting information can be downloaded at: https://www.mdpi.com/article/10.3390/life13041043/s1, Table S1: Stratified and total pooled random-effect estimates of the proportion of drop-outs due to any adverse event and stratification by diagnosis. % W is the percentage weight of each study; Table S2: Stratified and total pooled random-effect estimates of the proportion of drop-outs due to any reasons and stratification by diagnosis; Table S3: Stratified and total pooled random-effect estimates of the proportion of (hypo)mania switch and stratification by diagnosis.
