# Supplementary material for: Pramipexole Augmentation for Treatment-Resistant Unipolar and Bipolar Depression in the Real World: A Systematic Review and Meta-Analysis
Source: Life (Basel). 2023 Apr 19;13(4):1043. doi: 10.3390/life13041043 (PMC10141126; doi:10.3390/life13041043)
Supplement: Supplementary file 1 [file life-13-01043-s001.zip › life-2227256-supplementary.pdf]

Supplementary Table S1. Stratified and total pooled random-effect estimates of the proportion of drop-outs due to any adverse event and stratification by diagnosis. % W is the percentage weight of each study

Drop-outs due to any adverse event	**Events (n)**	**Patients (n)**	**Proportion**	**95%-CI**		**%W (random)**
**Unipolar**						
Fawcett et al. 2016 [85]	5	24	20.8%	7.1%	42.2%	6.3
Hori et al., 2012 [49]	0	12	0.0%	0.0%	26.5%	15.2
Inoue et al., 2010 [81]	2	10	20.0%	2.5%	55.6%	2.7
Sporn et al., 2000 [84]	1	20	5.0%	0.1%	24.9%	18.2
Tundo et al., 2022 [86]	5	79	6.3%	2.1%	14.2%	57.6
Random-effect			***6.4%***	***2.3%***	***10.5%***	
I^2, %			*30.0*			
**Bipolar**						
Fawcett et al. 2016 [85]	3	18	16.7%	3.6%	41.4%	16.9
Hori et al., 2012 [49]	0	5	0.0%	0.0%	52.2%	10.2
Sporn et al., 2000 [84]	3	12	25.0%	5.5%	57.2%	8.3
Tundo et al., 2022 [86]	3	37	8.1%	1.7%	21.9%	64.6
Random-effect			***10.1%***	***3.1%***	***17.2%***	
I^2, %			*0.0*			
**Total**						
Cassano et al., 2004 [80]	5	23	21.7%	7.5%	43.7%	9.4
Fawcett et al. 2016 [85]	8	42	19.1%	8.6%	34.1%	14.1
Hori et al., 2012 [49]	0	17	0.0%	0.0%	19.5%	19.7
Inoue et al., 2010 [81]	2	10	20.0%	2.5%	55.6%	5.4
Lattanzi et al., 2002 [82]	5	31	16.1%	5.5%	33.7%	12.9
Sporn et al., 2000 [84]	4	32	12.5%	3.5%	29.0%	14.6
Tundo et al., 2022 [86]	8	116	6.9%	3.0%	13.1%	23.9
Overall random-effects			***11.4%***	***5.0%***	***17.8%***	
I^2, %			*54.9*			

Supplementary Table S2. Stratified and total pooled random-effect estimates of the proportion of drop-outs due to any reasons and stratification by diagnosis.

Drop-outs due to any reasons	**Events (n)**	**Patients (n)**	**Proportion**	**95%-CI**		**%W (random)**
**Unipolar**						
Hori et al., 2012 [49]	2	12	16.7%	2.1%	48.4%	22.8
Inoue et al., 2010 [81]	2	10	20.0%	2.5%	55.6%	19.9
Sporn et al., 2000 [84]	9	20	45.0%	23.1%	68.5%	22.2
Tundo et al., 2022 [86]	6	79	7.6%	2.8%	15.8%	35.2
Random-effect			***20.4%***	***4.1%***	***36.7%***	
I^2, %			*73.8*			
**Bipolar**						
Hori et al., 2012 [49]	0	5	0.0%	0.0%	52.2%	17.1
Sporn et al., 2000 [84]	3	12	25.0%	5.5%	57.2%	13.9
Tundo et al., 2022 [86]	5	37	13.5%	4.5%	28.8%	69.0
Random-effect			***12.8%***	***3.7%***	***22.0%***	
I^2, %			*11.3*			
**Total**						
Cassano et al., 2004 [80]	14	23	60.9%	38.5%	80.3%	13.0
Fawcett et al. 2016 [85]	15	42	35.7%	21.6%	52.0%	15.0
Hori et al., 2012 [49]	2	17	11.8%	1.5%	36.4%	14.7
Inoue et al., 2010 [81]	2	10	20.0%	2.5%	55.6%	11.3
Lattanzi et al., 2002 [82]	12	31	38.7%	21.9%	57.8%	14.1
Sporn et al., 2000 [84]	12	32	37.5%	21.1%	56.3%	14.2
Tundo et al., 2022 [86]	11	116	9.5%	4.8%	16.3%	17.6
Overall random-effects			***29.7%***	***16.2%***	***43.3%***	
I^2, %			*86.6*			

Supplementary Table S3. Stratified and total pooled random-effect estimates of the proportion of (hypo)mania switch and stratification by diagnosis.

(Hypo)mania switch	Events (n)	Patients (n)	Proportion	95%-CI		%W (random)
**Unipolar**						
Cassano et al., 2004 [80]	1	11	9.1%	0.2%	41.3%	0.8
Fawcett et al. 2016 [85]	0	24	0.0%	0.0%	14.3%	7.8
Hori et al., 2012 [49]	0	12	0.0%	0.0%	26.5%	2.2
Inoue et al., 2010 [81]	0	10	0.0%	0.0%	30.9%	1.6
Lattanzi et al., 2002 [82]	0	14	0.0%	0.0%	23.2%	2.9
Sporn et al., 2000 [84]	0	20	0.0%	0.0%	16.8%	5.6
Tundo et al., 2022 [86]	0	79	0.0%	0.0%	4.6%	79.2
Random-effect			***0.1%***	***0.0%***	***1.6%***	
I^2, %			*0.0*			
**Bipolar**						
Cassano et al., 2004 [80]	2	12	16.7%	2.1%	48.4%	3.1
Fawcett et al. 2016 [85]	0	18	0.0%	0.0%	18.5%	27.0
Hori et al., 2012 [49]	0	5	0.0%	0.0%	52.2%	2.9
Lattanzi et al., 2002 [82]	2	17	11.8%	1.5%	36.4%	6.0
Perugi et al., 2001 [83]	1	10	10.0%	0.3%	44.5%	4.0
Sporn et al., 2000 [84]	1	12	8.3%	0.2%	38.5%	5.7
Tundo et al., 2022 [86]	1	37	2.7%	0.1%	14.2%	51.2
Random-effect			***3.5%***	***0.0%***	***7.2%***	
I^2, %			*0.0*			
**Total**						
Cassano et al., 2004 [80]	3	23	13.0%	2.8%	33.6%	1.0
Fawcett et al. 2016 [85]	0	42	0.0%	0.0%	8.4%	18.6
Hori et al., 2012 [49]	0	17	0.0%	0.0%	19.5%	3.3
Inoue et al., 2010 [81]	0	10	0.0%	0.0%	30.9%	1.3
Lattanzi et al., 2002 [82]	2	31	6.5%	0.8%	21.4%	2.6
Perugi et al., 2001 [83]	1	10	10.0%	0.3%	44.5%	0.6
Sporn et al., 2000 [84]	1	32	3.1%	0.1%	16.2%	5.3
Tundo et al., 2022 [86]	1	116	0.9%	0.0%	4.7%	67.5
Overall random-effects			***1.1%***	***0.0%***	***2.5%***	
I^2, %			*0.0*			
